# Comparison of perinatal outcomes in facilities before and after Global Network’s Helping Babies Breathe Implementation Study in Nagpur, India

**DOI:** 10.1186/s12884-019-2480-7

**Published:** 2019-09-04

**Authors:** Archana Patel, Akash Bang, Kunal Kurhe, Savita Bhargav, Amber Prakash, Spoorthy Arramraj, Patricia L. Hibberd

**Affiliations:** 1 Lata Medical Research Foundation, Nagpur, India; 2 Mahatma Gandhi Institute of Medical Sciences, Sewagram, Maharashtra State 442102 India; 3 Ankura Hospital for Women and Children, Hyderabad, India; 4 Boston University School of Public Health, Boston, MA USA

**Keywords:** Helping babies breathe, Asphyxia, Neonatal mortality, Perinatal mortality, Stillbirths, Essential newborn care, Training, Pre-post design, Facility births, Community births, Implementation science, Global Health, India

## Abstract

**Background:**

The Helping Babies Breathe (HBB) educational program focuses on training of first-level birth attendants in neonatal resuscitation skills for the first minute of life (The Golden Minute). Pre-post studies of HBB implementation in sub-Saharan Africa and Asia have shown reductions in facility-based very early neonatal mortality and stillbirth rates. However, the Global Network pre-post HBB Implementation Study (GN-HBB-IS) found no difference in day 7 perinatal mortality rates (PMR-D7) among births to women participating in the Global Network’s Maternal and Newborn Health Registry. To address potential differences in perinatal outcomes in births occurring in facilities that implemented HBB vs. all births occurring in the communities served by facilities that implemented HBB, we compared day-1 perinatal mortality rates (PMR-D1) among births occurring pre and post HBB implementation in facilities in Nagpur, India, one of the 3 sites participating in the GN-HBB-IS.

**Methods:**

We hypothesized that there would be a 20% decrease in the Nagpur facility based PMR-D1 in the 12 months post GN HBB implementation from the pre-period. We explored pre-post differences in stillbirth rates (SBR) and day-1 neonatal mortality rates (NMR-D1).

**Results:**

Of the 15 facilities trained for the GN-HBB-IS, 13 participated in the Nagpur HBB Facility Study (Nagpur-HBB-FS). There were 38,078 facility births in the 12 months before the GN-HBB-IS and 40,870 facility births in the 12 months after the GN-HBB-IS. There was 11% overlap between the registry births analyzed in the GN-HBB-IS and the facility births analyzed in the Nagpur-HBB-FS. In the Nagpur-HBB-FS, there was a pre-post reduction of 16% in PMR-D1 (*p* = 0.0001), a 14% reduction in SBR (*p* = 0.002) and a 20% reduction NMR-D1 (*p* = 0.006).

**Conclusions:**

In the Nagpur-HBB-FS, PMR-D1, stillbirths and NMR-D1 were significantly lower after HBB implementation. These benefits did not translate to improvements in PMR-D7 in communities served by these facilities, possibly because facilities in which HBB was implemented covered an insufficient proportion of community births or because additional interventions are needed after day 1 of life. Further studies are needed to determine how to translate facility-based improvements in PMR-D1 to improved neonatal survival in the community.

**Trial registration:**

The Global Network HBB Implementation Study (GN-HBB-IS) was registered at ClinicalTrials.gov: NCT01681017.

**Electronic supplementary material:**

The online version of this article (10.1186/s12884-019-2480-7) contains supplementary material, which is available to authorized users.

## Background

Neonatal deaths now account for around 46% of the under-5-years-old deaths and must be addressed to accelerate progress towards Sustainable Development Goal 3 (SDG3) [1–4], because one of the targets under SDG3 is to reduce the neonatal mortality to 12 per 1000 livebirths by 2030 [5]. While neonatal deaths have fallen from 5 to 2.5 million from 1990 to 2017, the annual rate of reduction in neonatal mortality over this period (2.6% per year) is much lower than that for children aged 1–59 months (3.7%) [1]. Among live-born babies, the risk of death is greatest on the first day of life–about 1 million deaths a year and 36% of all neonatal deaths [2]. Many of these deaths are caused by birth asphyxia or failure to establish breathing at birth. Excluded from these deaths are the estimated 1.3 million intrapartum or “fresh” stillbirths [6] who are not breathing at birth and for some reasons, are subjected to either inadequate or no resuscitation at all. For all these reasons, there is an intense global focus on interventions for the estimated 10 million babies requiring assistance to initiate breathing [7, 8].

Around 85% of babies born at term initiate spontaneous respiration within 10 to 30 s of birth, an additional 10% require initial steps such as tactile stimulation or airway clearing or positioning [9–13] and approximately 3% require positive-pressure ventilation by bag and mask [11, 13–15]. Training in these basic resuscitation measures should be able to salvage 90% of the babies that do not initiate spontaneous respiration and is most needed in low resource settings where there is inadequate access to intrapartum and immediate post-partum care [13, 16, 17]. Helping Babies Breathe (HBB) is a simple hands-on training curriculum in basic newborn resuscitation for birth attendants [18]. The training focuses on appropriate resuscitation skills to be used within the first 60 s of life (the Golden Minute) including timely delivery of the essential interventions such as drying, providing warmth, and clearing the airway, providing additional stimulation to breathe and bag and mask ventilation, if needed. In past, studies have attempted to quantify the impact of resuscitation on new-born outcome and have shown the benefits of resuscitation training on newborn outcomes, including the Bang et al. study in which resuscitation training in India reduced the asphyxia-related mortality by 65% (*p* < 0.02, [19]). Carlo et al. used a “train-the-trainer” model to sequentially train midwives in urban, community health clinics in Zambia in Essential Newborn Care (ENC) and in the American Academy of Pediatrics Neonatal Resuscitation Program (NRP), and found a decrease in the all-cause 7-day neonatal mortality rates from 11.5 to 6.8 deaths per 1000 live births after ENC training, which was further lowered by NRP training. Recently the Eunice Kennedy Shriver National Institute of Child Health and Human Development (NICHD) Global Network for Women’s and Children’s Health Research, and Research Triangle International (RTI) as the data coordinating center, conducted a pre-post study to evaluate the impact of HBB implementation in facilities serving rural communities (Maternal and Newborn Health Registry (MNHR)) located at three Global Network research sites - Nagpur and Belgaum in India and Eldoret in Kenya [18, 20]. MNHR enrolls pregnant women and records maternal and neonatal outcomes in catchment areas of rural primary health centers (study clusters). An additional file shows the flow diagram for the Nagpur Site of the Global Network HBB Implementation Study (GN-HBB-IS) [See Additional file 1]. All the births in the community based MNH registry were analyzed in the GN-HBB-IS. Around 45% of these did deliver in HBB trained facilities but rest delivered outside in other facilities. GN-HBB-IS did not find any effect of HBB implementation on day 7 perinatal mortality (PMR-D7). There are two plausible reasons for the failure to observe a reduction in PMR-D7 after facility-based health workers were trained in HBB implementation. Firstly, the reduction in PMR-D7 was estimated in all deliveries of pregnant women enrolled from the communities served by the trained facilities, not just those who delivered in HBB trained facilities. Secondly, the pre training period overlapped with the period of HBB training that was carried out in a step wise manner across the facilities and could have already started to reduce PMR-D7 in the pre-HBB implementation period (Table 1 and Additional file 1).

**Table 1:** Comparison between the Nagpur-HBB-FS and the Nagpur Site of the GN-HBB-IS

Criteria	Nagpur HBB Facility Study (Nagpur-HBB-FS)	Nagpur Site of the GN HBB Implementation Study (GN-HBB-IS – Nagpur)
Study Population	• All births in 13 of the 15 facilities that had participated in the GN-HBB-IS whether they belonged to the MNH registry area (around 11%) or not.	• All births in the GN MNH Registry whether delivered at facilities that participated in the GN-HBB-IS (around 45%) or at other facilities that did not receive HBB training and implementation.
Inclusion Criteria	• All stillbirths included	• Only fresh stillbirths included
Exclusion Criteria	• Miscarriage • Medical Termination of Pregnancy (MTP)	• Miscarriage • Medical Termination of Pregnancy (MTP) • Birth weight < 1500 g • Missing birth weight • Macerated stillbirths
Facility HBB Training Period	June 2012 to October 2012	
Pre HBB data collection Timing	• April 2011 – March 2012	• November 2011–October 2012
Post HBB data collection Timing	• November 2012–October 2013	• November 2012–October 2013
Outcomes	• PMR-D1 – (All stillbirths + day 1 neonatal mortality) • All stillbirths • NMR-D1 (Day 1 neonatal mortality)	• PMR-D7 (only fresh stillbirths + day 7 neonatal mortality) • Only fresh stillbirths • PMR-D1 (only fresh stillbirths + day 1 neonatal mortality)
Data Source	• Pre-existing standard facility records like birth and mortality registers	• GN MNH Registry data collection forms

Since the GN-HBB-IS was not designed to evaluate facility based changes in day 1 perinatal mortality (PMR-D1), we planned this facility based study (Nagpur-HBB-FS) and collected data from the Nagpur facilities participating in GN-HBB-IS before any training had commenced and after the GN-HBB-IS training had been completed for all the births in the facilities irrespective of whether or not they belonged to the MNH registry. Our objective was to evaluate PMR-D1 in births occurring in facilities pre- compared to post- GN-HBB-IS implementation.

## Methods

### Study site for the facility based study

This study (The Nagpur-HBB-FS) was conducted and coordinated by the research foundation located at Nagpur, which is a Global Network site [20]. In this study we included 13 of the 15 facilities that had participated in the GN-HBB-IS as two tertiary level facilities declined consent for sharing their pre-training data. The 13 facilities that participated in the Nagpur-HBB-FS included 2 primary facilities (facilities where caesarian section facility is not available), 4 secondary facilities (where caesarian section facility is available on call) and 7 tertiary facilities (where caesarian section facility is available round the clock along with allied emergency services). All the facilities except the tertiary facilities were located in rural areas. The primary, secondary and tertiary facilities had 874, 2882, and 34,322 annual deliveries respectively in the pre-HBB period. Their pre-HBB baseline perinatal mortality rates per 1000 births were 13.73, 23.95, and 28.79 respectively. None of the facilities were providing a structured ENC and HBB training prior to the study.

### Implementation of HBB training in facilities

The training for GN-HBB-IS followed the approach developed by the American Academy of Pediatrics and has been described in detail elsewhere [18, 20, 21]. Briefly, it consisted of the training of Master Trainers (MT) who then trained facility birth attendants (BAs) in HBB; the introduction of a multi-faceted monitoring program; and retraining of the active BAs after six months. BAs were instructed to resuscitate all non-macerated births, including those considered fresh stillbirths.

Standard HBB training materials and equipment (Laerdal NeoNatalie® equipment and materials and clean delivery kits) were provided to all facilities and MTs based on delivery volume at the time of training – these materials and equipment were not available in the pre-study period. New BA recruits who joined the facility after the initial training were also provided HBB training immediately on recruitment. All the staff of the health facilities were also trained in basic Essential Newborn Care (ENC) [22].

The initial Master Trainers (MT) training workshop was held in May 2012. The Nagpur MT then conducted facility level training workshops for birth attendants (BAs) between June and October 2012. Refresher trainings were conducted six months after the initial HBB training.

### Monitoring of HBB implementation in facilities

Monitoring activities were introduced after the initial facility-level training of BAs and included daily bag and mask ventilation practice sessions when BAs reported for work and signed the logbooks; daily checks of availability, cleanliness, and function of resuscitation equipment; regular observation of deliveries; debriefing after every resuscitation; audits of every perinatal death; once a month quality assurance (QA) visits to observe HBB skills directly during deliveries or test the BAs using a neonatal simulator if no deliveries were available [18, 20, 21]. Monthly monitoring reports were reviewed and bi-weekly data review calls were made with the central core staff (RTI International and NICHD), followed by feedback to facility MTs and BAs.

### Study outcomes for the HBB trained facilities in Nagpur, India

Our primary hypothesis for the Nagpur-HBB-FS was that here would be at a 20% decrease in Nagpur facility based PMR-D1 in the 12 months pre vs. post training and implementation period. PMR-D1 included stillbirths or within 24 h of birth. We explored pre-post differences in stillbirth rates (SBR) and in day-1 neonatal mortality rates (NMR-D1).

Our primary outcome was PMR-D1 pre-post GN-HBB-IS. Exploratory outcomes included pre-post GN-HBB-IS SBR (fresh and macerated stillbirths per 1000 total births) and NMR-D1 per 1000 live births. We collected one-year pre training data (April 2011 to March 2012) from the pre-existing standard hospital records like birth registers and mortality registers of the Nagpur facilities participating in GN-HBB-IS. The training was conducted from June 2012 to October 2012. The post GN-HBB-IS training data (November 2012 to October 2013) was also collected from similar pre-existing standard hospital records- different from the data collection tools used in the GN-HBB-IS.

In the pre- period, stillbirths were not routinely categorized as fresh or macerated, even though the facilities were trained to make this distinction during the GN-HBB-IS study and in the post period. For this reason, in both periods, we combined fresh and macerated stillbirths and reported them as total stillbirths. Similarly, PMR-D1 was defined as fresh or macerated stillbirths plus deaths within 24 h of birth. NMR-D1 was defined as neonatal death within 24 h of a live birth.

### Data management

Primary and secondary outcomes were obtained from the facility medical records by trained data collectors. The following data was collected for pre -post period- number of live births, mode of delivery, presence of multiple gestations, gender of baby and presence of maternal complications (gestational diabetes mellitus, pregnancy induced hypertension, abruptio placentae, eclampsia, sickle cell disease, or other chronic illness) for all PMR-D1.

### Power and statistical analysis

We assumed that the facility based pre GN-HBB-IS PMR-D1 would be 25/1000 total births, based on historical data from the facilities. If total births occurring in the year before and after GN-HBB-IS was greater than 19,000 in both periods, the study would have greater than 90% power to detect a 20% reduction in pre to post PMR-D1 (Sample size estimates were obtained using nQuery Advisor version 7.0, Statistical Solutions, Boston, 2012).

We compared the primary and exploratory outcomes pre and post the GN-HBB-IS using the Fisher Normal Test. We also used multilevel mixed effects Poisson modeling to predict PMR-D1 based on neonatal and maternal characteristics. The level of care of facility (primary, secondary or tertiary) and time (“pre-HBB period” or “post-HBB period”) were fixed effects in the model and the 13 participating facilities were included as random effects to obtain robust standard errors.

### Ethical approval

The study protocol for the Nagpur-HBB-FB study was reviewed and approved by the Institutional Review Board of Lata Medical Research Foundation, Nagpur. All facility-based data was obtained from medical records so no specific consent was obtained from the women giving birth at the facilities.

## Results

**Fig. 1:**
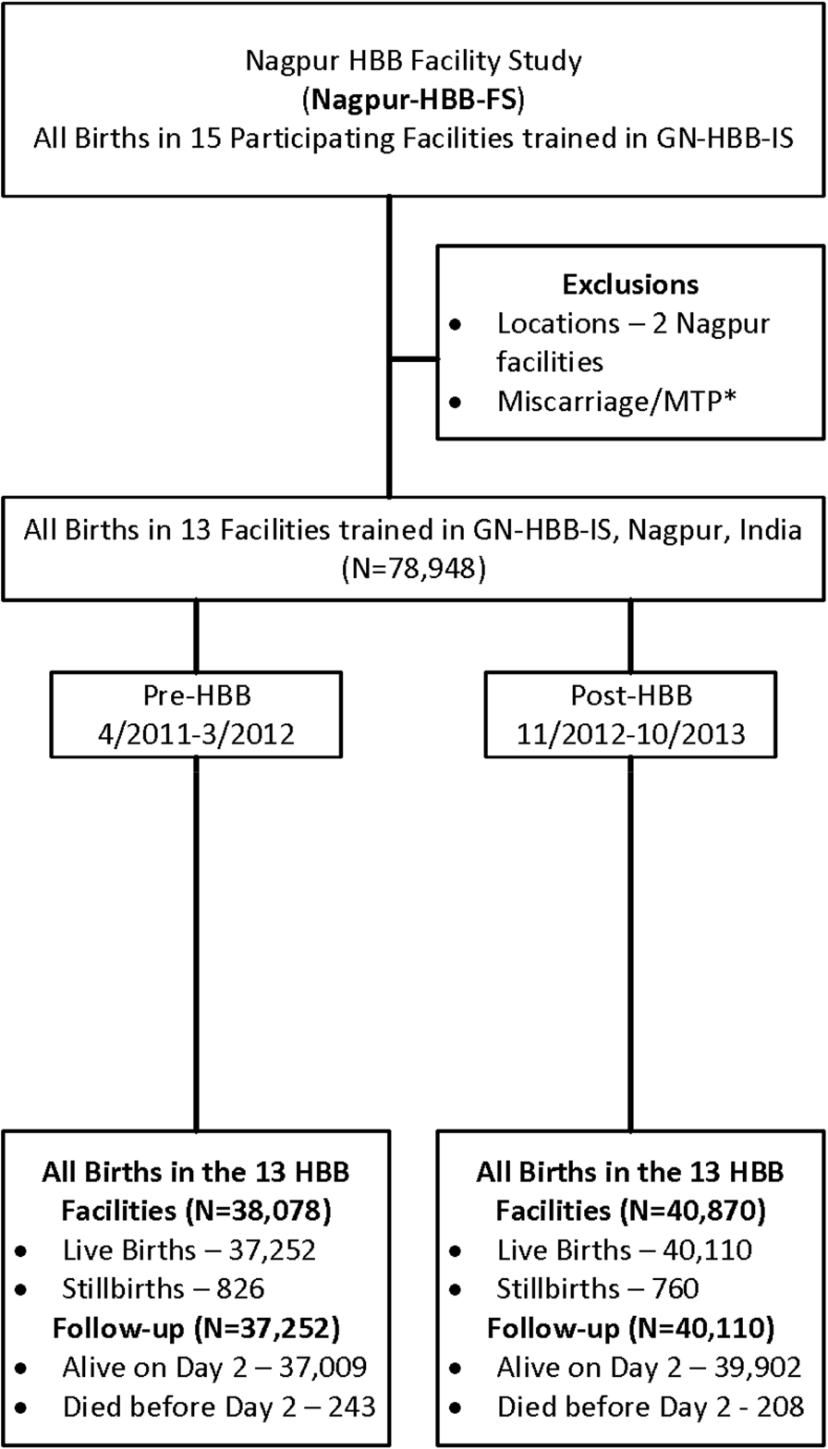
Flow Diagram for the Nagpur, India HBB Facility Study (Nagpur-HBB-FS)

**Fig. 2:**
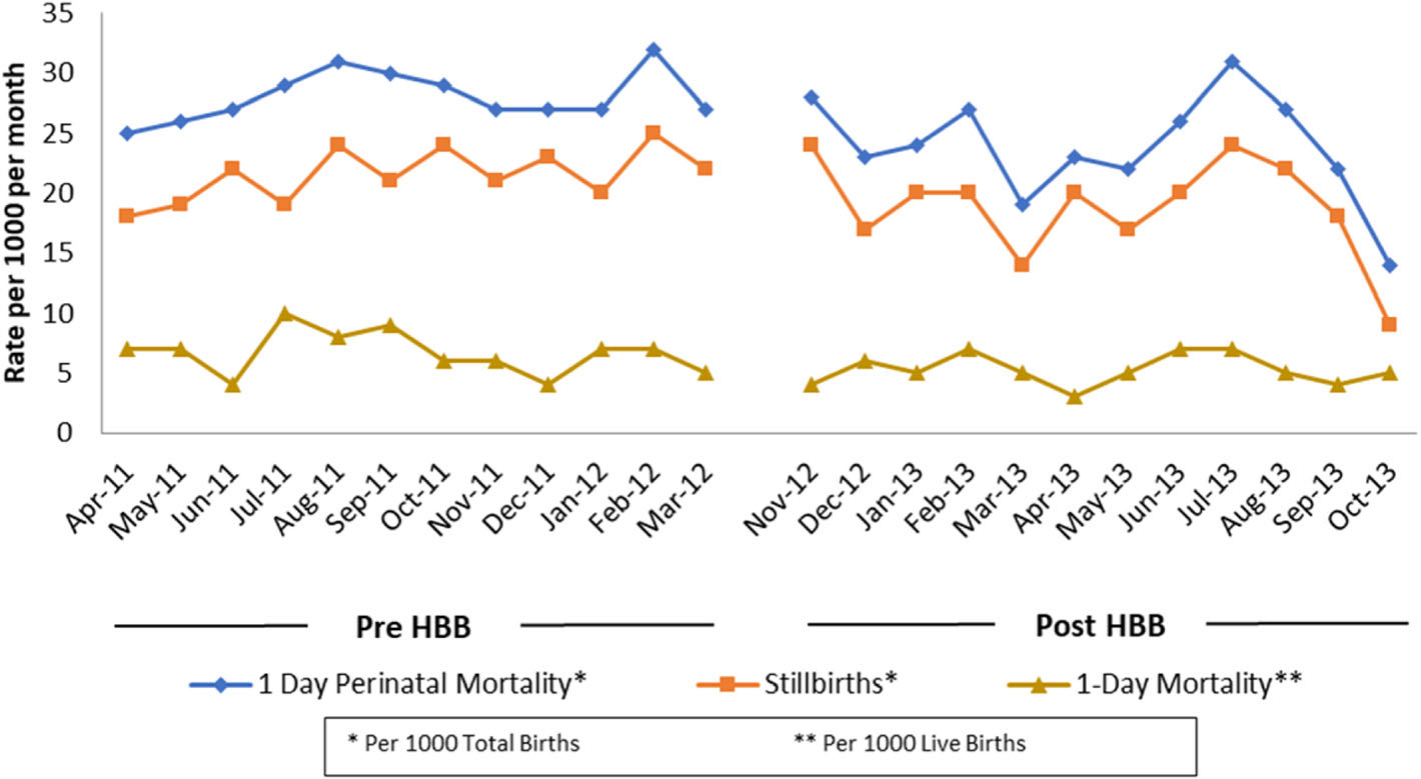
Nagpur-HBB-FS Monthly trends in 1-day Perinatal Mortality Rates

As shown in the flow diagram in Fig. 1, there were 78,948 births in the 13 HBB facilities in Nagpur – 38,078 in the pre-HBB and 40,870 in the post-HBB period. The majority of these births (71,489) took place in tertiary care facilities, 1731 took place in primary and 5728 in secondary facilities. Table 2 shows PMR-D1, SBR and NMR-D1 pre- and post-HBB, all of which were significantly reduced in the post-HBB period. The model yielded a significant incidence risk ratio (IRR) post to pre HBB of 0.85 (95% CI 0.73–0.98) for PMR-D1, of 0.86 (95% CI: 0.76–0.98) for SBR and of 0.79 (95% CI: 0.62–1.00) for NMR-D1. Figure 2 shows monthly PMR-D1, SBR and NMR-D1 pre- and post- HBB in the 13 facilities, all of which were significantly lower in the post vs. pre HBB period.

**Table 2:** Nagpur-HBB-FS Outcomes

Outcome	Pre HBB		Post HBB		p [95% CI for diff.)
	*n*	Rate	*n*	Rate	
1-Day Perinatal Mortality (PMR-D1)	1069	28.07^a^	968	23.68^a^	0.0001 [2.1,6.6]
Stillbirths (SB)	826	21.69^a^	760	18.6^a^	0.002 [1.1, 5.0]
1-Day Mortality (NMR-D1)	243	6.52^b^	208	5.18^b^	0.006 [0.5, 3.6]

^a^Calculated by total births in the denominator - for pre HBB, *N* = 38,078; for post HBB *N* = 40,870

^b^Calculated by live births in the denominator - for pre HBB, *N* = 37,252; for post HBB *N* = 40,110

Among the PMR-D1, there was no significant difference in the pre vs post period for the baseline maternal and neonatal characteristics such as caesarian sections (10.8% vs 12.8%), prematurity (66.3% vs 68.2%), male gender (54.5% vs 53.2%), multiple gestations (7.3% vs 6.0%) and low birth weight (83.3% vs 86.8%). Maternal complications were present in 15.3% in pre and 11.1% in post period (p: 0.004).

## Discussion

This study showed that by focusing on day 1 perinatal and neonatal mortality just in trained facilities in one of the three GH-HBB-IS sites in Nagpur, India, HBB implementation significantly reduced PMR-D1 by 15.6%, SBR by 14.2% and NMR-D1 by 20.6%. By contrast, the NICHD GN-HBB-IS did not result in improvements in PMR-D7 in communities that were partially served by the facilities in which staff were trained in the GN-HBB-IS in the Nagpur or other GN sites [20]. There could be two likely reasons for this discrepancy. Firstly, there was an overlap of pre-HBB period and the training period in GN-HBB-IS which may have reduced PMR-D7 during the pre-period and may have led to the null result. Secondly, the GN-HBB-IS analyzed all births in a community-based registry. Around 45% of these births did take place in HBB trained facilities but the rest (more than 50% of babies) delivered outside in other facilities that were not trained in HBB implementation. Consequently, where the intervention did not happen, there was likely no effect and this diluted the effect in the overall population. In contrast the Nagpur-HBB-FS study analyzes ALL facility births- whether or not they belonged to the MNH registry- from 13 of the 15 facilities that had participated in the GN-HBB-IS. Since around 11% of these facility births belonged to the MNH registry, there was about 11% overlap in the births analyzed in GN-HBB-IS and Nagpur-HBB-FS (See Table 1, Fig. 1, and Additional file 1).

Similar to other studies, we also found a significant reduction in the SBR post HBB implementation, likely because HBB improves identification and resuscitation of neonates that appear to be lifeless at birth [23–25].

The main strength of our Nagpur-HBB-FS is that it evaluated the direct impact of HBB implementation (HBB Training, equipment’s, and monitoring activities as explained in methods) with in a range of primary care facilities (where birth attendants may have minimal training) to tertiary care facilities (with high patient volumes and likely more complicated deliveries). Important limitations include lack of information on whether the study population changed in the pre to post period and the impact of HBB training on how perinatal mortality and stillbirths were recorded in the medical record in the two periods. Also, since many newborns get discharged from facilities before 7 days, in absence of any follow up information on the newborn’s status at home, we were unable to report PMR-D7 in the Nagpur-HBB-FS to assess whether the improvements seen in PMR-D1 were sustained through day 7.

So, where does this analysis fit in our understanding of the role of HBB implementation in neonatal resuscitation? Our study suggests that HBB improves PMR-D1 but may not translate to subsequent neonatal survival as the impact of neonatal resuscitation trainings on reduction of neonatal deaths will be highest for 1-day deaths and diminish subsequently. This fact has been endorsed in a recent meta-analysis of 20 studies that assessed impact of neonatal resuscitation trainings which showed a 42% reduction in 1-day mortality and 18% reduction in 7-day mortality [26]. Reduction of 28-days neonatal mortality will thus require, in addition to resuscitation trainings, provision of specialized care to the resuscitation survivors and various other existing intervention packages like improved hygiene at birth, breastfeeding and simple approaches to keep babies warm etc. [27, 28] as predominant causes of mortality after 24 h of life are prematurity, hypoxic ischemic encephalopathy, sepsis and congenital malformations [29, 30]. The GN-HBB-IS provides a bold target for where we need to go to improve global neonatal survival. Focus on facilities alone may not be sufficient to translate the gains achieved in neonatal survival to be generalized to population level gains.

## Conclusion

This study addresses differences between facility based perinatal mortality rates on day 1 of life and community based perinatal mortality rates through day 7 of life. In this study (Nagpur-HBB-FS), facility based SBR, NMR-D1, and PMR-D1 were significantly lower after HBB implementation. In the previously published study (GN-HBB-IS), these facility based benefits did not translate to improvements in PMR-D7 in communities served by these facilities, possibly because all the community births did not occur in the facilities where HBB was implemented or because additional interventions are needed after day 1 of life. Benefits of HBB may thus be limited if births do not occur in facilities where HBB training has been provided. Increased access to facilities with HBB training will reduce perinatal mortality in that community.

## Additional file

Additional file 1: Flow Diagram for the Nagpur Site of the Global Network HBB Implementation Study. (DOCX 72 kb)

## Data Availability

A de-identified minimal dataset for the Nagpur-HBB-FS can be shared upon reasonable request to the corresponding author. All authors hereby declare that individual participant data, in any form is not a part of this publication.

## Abbreviations

BAs: Birth Attendants
ENC: Essential Newborn Care
GN: Global Network for Women’s and Children’s Health Research
GN-HBB-IS: Global Network Helping Babies Breathe Implementation Study
HBB: Helping Babies Breathe
MT: Master Trainer
MTP: Medical Termination of Pregnancy
Nagpur-HBB-FS: Nagpur Helping Babies Breathe Facility Study
NICHD: *The Eunice Kennedy Shriver* National Institute of Child Health and Human Development
NMR: Neonatal Mortality Rate
NRP: Neonatal Resuscitation Program
PMR: Perinatal Mortality Rate
RTI: Research Triangle Institute
SBR: Stillbirth Rate
