# Supplementary material for: Comparison of perinatal outcomes in facilities before and after Global Network’s Helping Babies Breathe Implementation Study in Nagpur, India
Source: BMC Pregnancy Childbirth. 2019 Sep 4;19:324. doi: 10.1186/s12884-019-2480-7 (PMC6724302; doi:10.1186/s12884-019-2480-7)

**Additional File 1:** Flow Diagram for the Nagpur Site of the Global Network HBB Implementation Study


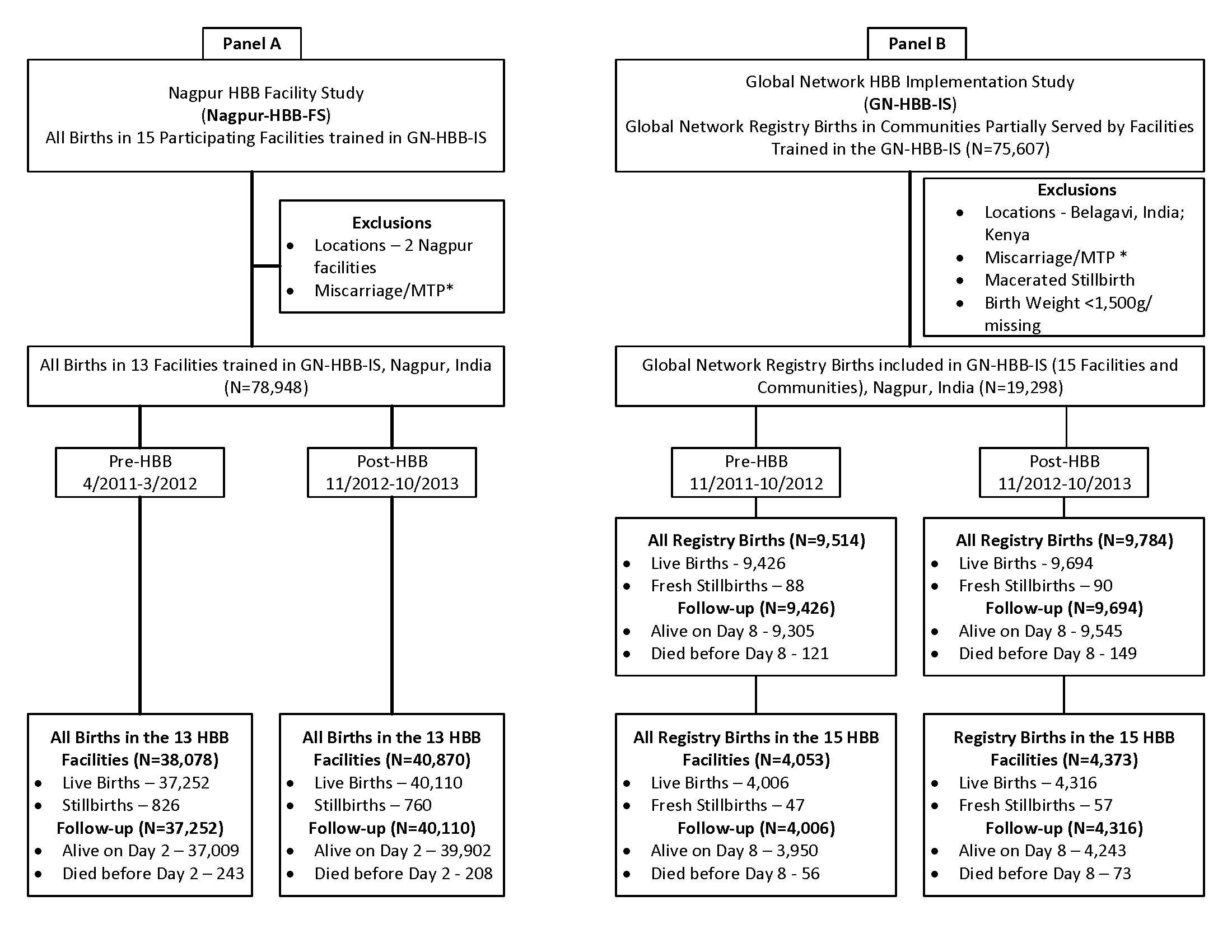

Supplement: Supplementary file 1 — Flow Diagram for the Nagpur Site of the Global Network HBB Implementation Study. (DOCX 72 kb) [file 12884_2019_2480_MOESM1_ESM.docx]
